# Exploring the therapeutic mechanism of itraconazole combined with ritonavir on *Candida albicans* infection through network pharmacology and molecular docking

**DOI:** 10.3389/fphar.2025.1578749

**Published:** 2025-07-14

**Authors:** Yiyang Feng, Wenli Feng, Jing Yang, Yan Ma

**Affiliations:** Department of Dermatovenereology, The Second Hospital, Shanxi Medical University, Taiyuan, Shanxi, China

**Keywords:** *Candida* albicans, itraconazole, ritonavir, autophagy, network pharmacology

## Abstract

**Background:**

Autophagy induced by itraconazole and ritonavir was found involved in the pathogenesis of *C. albicans*. This study was designed to explore the possible molecular mechanism of itraconazole and ritonavir in the treatment of *Candida albicans* infection through autophagy pathway.

**Methods:**

The overlapping targets of itraconazole and ritonavir, and those-related to *C. albicans* and autophagy were screened. Then the core targets were identified by protein-protein interaction (PPI) network analysis. Gene enrichment analysis of targets and the drug-target-pathway-disease network was constructed. The interactions between itraconazole, ritonavir and core targets were analyzed by molecular docking and molecular dynamics simulation. Finally, the core target-miRNA interaction network was constructed to predict candidate miRNAs.

**Results:**

PPI network showed that PIK3R1, RELA, STAT3, HSP90AA1, TP53, JUN, GRB2, EGFR, ESR1 and TNF were potential core targets of autophagy therapy for *C. albicans* infection with itraconazole and ritonavir. Enrichment analysis showed that the two drugs may regulate the autophagy process through pathways including PI3K-AKT, IL-17, MAPK, Toll-like receptor, JAK-STAT and NF-κB. Molecular docking analysis indicated that itraconazole and ritonavir possess strong binding affinities with the cote target proteins, with binding free energies ranging from −5.6 to −9.5 kcal/mol. Key interactions were identified at the active sites of the targets, suggesting stable ligand-receptor complex formation. Itraconazole docked to PIK3R1 through SER-78 and GLU-82 (−9.3 kcal/mol), and ritonavir docked to PIK3R1 through ASN-85, GLU-1011 and arginine (ARG)-1088 (−7.7 kcal/mol). Molecular dynamics simulation of itraconazole and ritonavir with representative target genes lasted for 100 ns showed the structures of the formed complexes remained stable throughout. Finally, the candidate miRNAs including miR-486-5p, miR-411-5p.1 and miR-296-5p were identified.

**Conclusion:**

Network pharmacological analysis showed a multi-target and multi-pathway molecular mechanism of itraconazole and ritonavir in the treatment of *C. albicans* infection, and provided a theoretical basis for subsequent studies.

## Introduction

As a common opportunistic fungus, *C. albicans* can result in skin, mucous membrane and even multiple system infections when the immune function is damaged. Candidemia caused by *Candida albicans* has an all-cause mortality of 25%–72% within 30 days (Doi et al., 2016; Lamoth et al., 2018; Tsay et al., 2020; Lopes and Lionakis, 2022; Agnelli et al., 2023). In recent years, with the widespread use of fungicide, reports about the resistance of *C. albicans* to azoles, polyenes, echinocandins, etc., have increased (Enoch et al., 2017; Burki, 2023; Cook et al., 2023). At the same time, there are rising numbers of newly diagnosed immunocompromised patients, causing an increased threat of *C. albicans* infection in healthy individuals (Enoch et al., 2017; Burki, 2023; Cook et al., 2023). Given the rising drug resistance of C. albicans and the increasing burden of immunocompromised populations, strategies involving the repurposing of conventional antifungals with synergistic activity offer a pragmatic solution. This approach not only circumvents the lengthy cycle and high costs of *de novo* drug development but also reduces therapeutic dosage and minimizes adverse reactions through combinatorial effects (Thompson et al., 2023).

Itraconazole is one of the triazoles used in the clinical treatment of *C. albicans* infection (Alyahya et al., 2023). Itraconazole has the advantages of broad spectrum and low side effects, but the drug-resistant strains have gradually increased, and the curative effect of monotherapy is subefficacious (Ye et al., 2022). Ritonavir has been used for more than 20 years as an HIV Protease inhibitor, moreover, there is increasing evidence that ritonavir has good anti-*C. albicans* activity. Our previous study also showed that ritonavir inhibited the L-aspartic acid activity of *C. albicans* in a dose-proportional manner (Feng et al., 2021). Therefore, combination therapy of *C. albicans* infection with itraconazole and ritonavir is a potentially feasible strategy to overcome the limitations of monotherapy.

Autophagy is not only involved in the pathogenesis of *C. albicans* infection, but also in mechanisms of antifungal defence (Lionakis et al., 2023). Wu et al. have indicated that upregulation of riboflavin metabolism and induction of mitochondrial dysfunction lead to increased autophagy, which can affect *C. albicans* virulence and lead to cell death in *C. albicans* (Wu et al., 2023). Autophagy participates in the rapid induction of neutrophil extracellular traps, which can trap and kill fungal hyphae that are too large for phagocytosis (Liang et al., 2022). Upregulated autophagy-related gene-3 can inhibit apoptosis in an autophagy-dependent manner, thereby mitigating tissue damage caused by *C. albicans* infection (Zheng et al., 2022). Autophagy-related proteins autophagy related 16 like 1 (ATG16L1) and ATG5 inhibit *C. albicans*-induced epithelial cell death early in *C. albicans* infection (Lapaquette et al., 2022). Itraconazole can induce autophagy-mediated cell death in colon cancer by Hedgehog signaling pathway (Deng et al., 2020). Ritonavir can induce autophagy in human liposarcoma cells (Gibellini et al., 2012). Autophagy of *C. albicans* and infected host cells was involved in the pathogenesis and therapy of *C. albicans* infection, but the mechanism of itraconazole and ritonavir in regulating autophagy in the treatment of *C. albicans* infection remains unclear.

MicroRNAs (miRNAs) play critical roles in post-transcriptional regulation of autophagy-related genes, with emerging evidence linking specific miRNAs to fungal infection and host immune responses (Croston et al., 2018; Xie et al., 2025). For instance, miR-199a has been shown to modulate autophagy via downregulating IFN-β expression in *Mycobacterium bovis* infected cells (Wang et al., 2018), while certain miRNAs have been related to host response to *C. albicans* infection, such as miR-155, miR-146, miR-455, miR-125a, miR-21-5p and miR-24-3p (Monk et al., 2010; Agustinho et al., 2017; Halder et al., 2021). However, the role of miRNAs in mediating the autophagy-dependent antifungal effects of itraconazole and ritonavir remains entirely unexplored.

Network pharmacology is commonly applied in the interactions between drugs and disease-related genes, analysis of the biological processes (BP) and signal pathways of gene enrichment, construction of the correlations among drugs, targets, pathways, and diseases, assistance in drug development and evaluation (Li et al., 2023). A previous report indicated that autophagy in HEPG2 cells has been found to be associated with cancer pathway and the PI3K-AKT signaling pathway (Cheng et al., 2022). Additionally, molecular docking has been applied to the screening and prediction of drugs or target genes (Fink et al., 2022). Molecular dynamics simulation assigns initial positions and velocities to each atom in the system and tracks their motion trajectories in three - dimensional space in real - time by accurately calculating the inter - atomic forces. This method can deduce dynamic processes such as molecular conformational changes, diffusion, and chemical reactions, and is widely applied in disease treatment and prevention research (Wu et al., 2022; Mu et al., 2025). Network pharmacology and molecular docking was conducted to systematically study the drug-target-pathway-disease network of itraconazole and ritonavir in combination with autophagy for the treatment of *C. albicans* infection, and to further clarify the mechanism of itraconazole combined with ritonavir in the regulation of autophagy in the therapy of *C. albicans* infection. This may provide reference for the clinical application of itraconazole combined with ritonavir in *C. albicans* infection.

## Methods

### Target prediction of itraconazole and ritonavir

Terms including “itraconazole”, “ritonavir”, “*C. albicans*”, and “autophagy” were searched in the Medical Subject Headings (MeSH) database. The MeSH terms were used to retrieve target predictions corresponding itraconazole and ritonavir in the DrugBank (https://www.drugbank.ca/), the Comparative Toxicogenomics Database (http://ctdbase.org/) (Davis et al., 2009), the SwissTargetPrediction (https://www.swisstargetprediction.ch) (Daina et al., 2019), the SuperPred (http://prediction.charite.de/), the SEA (https://sea.bkslab.org/), the TargetNet (http://targetnet.scbdd.com), and the PharmMapper (http://lilab.ecust.edu.cn/pharmmapper/index.php) databases (Wang et al., 2017). The predicted targets were merged to remove duplicates by the UniProt database (https://www.UniProt.org) with the Gene Name identifiers.

### Prediction of disease and autophagy-related targets

*C. albicans*-related targets were obtained from GeneCards (https://www.genecards.org/) (Safran et al., 2010), OMIM (https://omim.org/) (Amberger and Hamosh, 2017), PharmGKB database (https://www.pharmgkb.org) (Altman, 2007), and autophagy-related genes were retrieved from human autophagy database (HADb) (http://www.autophagy.lu/index.html), molecular signatures database (MSigDB) (https://www.gsea-msigdb.org/gsea/msigdb/), and GeneCards databases. The overlapping genes of targets related to itraconazole and ritonavir and those-related to *C. albicans* and autophagy were identified, and Venn diagrams were drawn using R 3.5.3. Those overlapping genes were used as potential targets for autophagy therapy of *C. albicans* infection in combination with itraconazole and ritonavir.

### Protein-protein interaction (PPI) network

The overlapping targets were uploaded to String 11.5 (https://STRING-db.org/) to construct a preliminary PPI network with the minimum interaction score = 0.900. Then the protein interactions were introduced into Cytoscape 3.8.2, and the degree topology algorithm in cytoHubba plugin was used to analyze the key targets and the PPI network of itraconazole and ritonavir for autophagy-related targets in the treatment of *C. albicans* infection was constructed. The top 10 targets with higher degree were chosen to be core targets.

### Gene Ontology (GO) and pathway enrichment analysis

The key targets were entered into the Metascape (http://Metascape.org/) database (Zhou et al., 2019) with a threshold of P < 0.01. GO function and Kyoto Encyclopedia of Genes and Genomes (KEGG) pathway enrichment analysis was performed. The top 20 pathways were selected for bubble diagram with R language.

### The drug-target-pathway-disease network

The KEGG pathway and the drug targets were mapped, and the results were imported into cytoscape 3.8.2 software to construct and visualized the drug-target-pathway-disease network.

### Molecular docking

The Protein Data Bank (PDB) ID of the receptor proteins encoded by the core target genes were searched in UniProt Database with resolution <3.0 Å, and the protein structures were downloaded from the Protein Database (http://www.rcsb.org/PDB/) (Burley et al., 2019). SDF files of structure of itraconazole and ritonavir were downloaded from the PubChem database. The energy of drug molecules was minimized using Chem3D. AutoDockTools (http://mgltools.scripps.edu/downloads) was utilized to remove the ligand and water molecules of the receptor protein, and the twisting bond of the drug was measured. Protein hydrogenation was performed using PyMOL (https://PyMOL.org/). Finally, AutoDock Vina Software (Trott and Olson, 2010) was used for docking, and PyMOL was used to visualize the best docking result.

### Molecular dynamics simulation

The protein - ligand combinations with lower binding energies from molecular docking were selected to perform 100 - ns molecular dynamics simulations based on GROMACS (Abraham et al., 2015). The simulation system uses the three - site rigid water molecule water model, and energy minimization as well as equilibrium procedures in the isothermal isovolumic ensemble (NVT) and isothermal isobaric ensemble (NPT) ensembles were carried out. Additionally, the root - mean - square deviation (RMSD), root - mean - square fluctuation (RMSF), radius of gyration (RoG), and solvent - accessible surface area (SASA) of the complexes were further analyzed (Sangavi et al., 2025). The results were visually by Qtgrace.

### Core target-miRNA interaction network and miRNA prediction

miRNAs corresponding to core targets were acquired in the TargetScan database (https://www.TargetScan.org/vert_80/) (McGeary et al., 2019). Then miRNAs whose seed match = 8 mer and conserved in mammals were screened. Cytoscape 3.8.2 was utilized to build the core target-miRNA interaction network. The most functional miRNAs that may be participated in autophagy-based treatment of *C. albicans* infection with itraconazole and ritonavir were predicted based on the Context score percentile values.

## Results

### The itraconazole, ritonavir, C. albicans and autophagy-related targets were obtained

**FIGURE 1:**
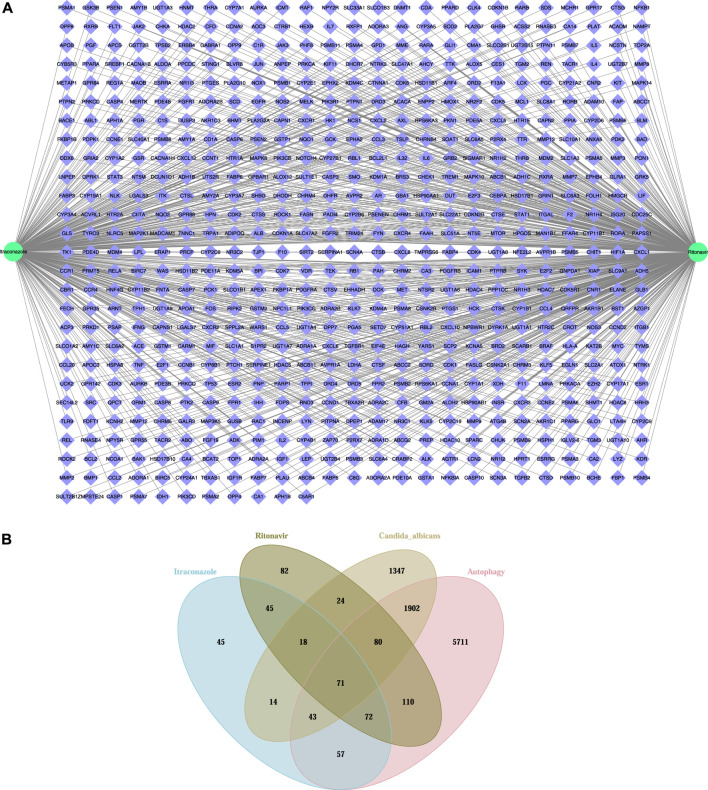
The predictive target genes of itraconazole and ritonavir **(A)**, and the Venn diagram showed the potential autophagy-related targets of itraconazole and ritonavir for treatment of *Candida albicans* infection **(B)**.

The target proteins of itraconazole and ritonavir were searched from UniProt and 365 target proteins were obtained for itraconazole, and 502 target proteins were obtained for ritonavir. Among these proteins, 661 targets were shared by itraconazole and ritonavir (Figure 1A). In addition, 3499 *C. albicans*-related targets and 8046 autophagy-related targets were retrieved from the UniProt database. By integrating these targets by Venn diagram, 194 potential autophagy-related targets of itraconazole and ritonavir for treatment of *C. albicans* infection were identified, including 80 genes overlapped by ritonavir targets, *C. albicans*-related targets, and autophagy-related genes, 71 genes overlapped by itraconazole targets, ritonavir targets, *C. albicans*-related targets, and autophagy-related genes, as well as 43 genes overlapped by itraconazole targets, *C. albicans*-related targets, and autophagy-related genes (Figure 1B, Table 1).

**TABLE 1:** The gene list targeted by itraconazole and ritonavir that are present in *Candida albicans*.

Gene list A	Gene list B	Gene list C
HDAC7	CXCR4	XDH
GRB2	SOD2	PDGFRB
UTS2R	CYP1A1	TYMS
PSMB8	CAPN1	EIF4E
CSNK2A1	CASP1	ACACA
GNPDA1	MME	SCN4A
SERPINA1	CCNA2	SCD
HK1	CTNNA1	PTPN1
HEXB	PADI4	PSAP
LYN	EGFR	ESRRA
IFNG	LGALS3	IL5
FOS	ABL1	ITGB1
DDX6	PGR	GLI1
CXCL1	PIK3R1	PIK3CD
LMNA	ALB	GABRA1
IL7	ERBB4	CYP17A1
IGF1	MMP13	GLS
ALDH2	CHEK1	P2RX7
HSPA8	ABCB1	RAC1
FDFT1	MAPK14	BLM
DHCR7	STAT3	CYP51A1
HDAC4	ANG	GRIA2
CXCR3	CNR2	NTRK1
PSMB4	NOS3	NOS2
LYZ	FGFR1	CCNE1
ABCC1	DHFR	ADH5
SREBF1	TNF	AHR
PSMA3	SIRT2	CYP19A1
EPHA2	ESR1	WARS1
FGF19	CDK4	CXCR1
RORB	STAT1	RIPK2
CDK6	HDAC8	KDM1A
FGFR2	GUSB	SIGMAR1
CHUK	CCL5	TLR9
CHKA	PRKACA	FYN
FAP	NFKB1	IL4
PRKD1	KCNH2	HSP90AB1
ICAM1	PPARG	MAN1B1
SORD	CXCL8	ADAM17
DYRK1A	CTSD	TGM2
MIF	HSP90AA1	ALK
CCL3	RARA	MTOR
TTK	NR1H4	SLC9A1
PRKCA	FDPS	
TOP1	ELANE	
MET	BCL2	
ROCK2	MAPK10	
NCSTN	NFE2L2	
MYC	CDK2	
CXCL2	CXCL10	
CAPN2	PDGFRA	
PDE10A	NFKBIA	
CDKN1A	ITGAL	
PRKCD	TGFB2	
SCARB1	CNR1	
IL6	MAOB	
FASLG	CTSG	
CXCL12	SYK	
RELA	IGF1R	
INSR	HIF1A	
SPARC	MAP2K1	
APOA1	CYP3A4	
JUN	F2	
CSNK2B	HMOX1	
FBP1	YARS1	
RB1	EPHB4	
TP53	FASN	
ZMPSTE24	IL2	
GLB1	PSMB1	
ESR2	DHODH	
CDK3	MMP9	
CYP1B1		
CASP8		
RAF1		
DRD2		
CCL20		
CCL2		
REL		
CDK5		
PSMB9		

Gene list A indicated genes that were overlapped by ritonavir targets, *Candida albicans*-related targets, and autophagy-related genes; Gene list B were genes that were overlapped by itraconazole targets, ritonavir targets, *Candida albicans*-related targets, and autophagy-related genes; Gene list C were genes that were overlapped by itraconazole targets, *Candida albicans*-related targets, and autophagy-related genes.

### PPI network establishment and core targets identification

**FIGURE 2:**
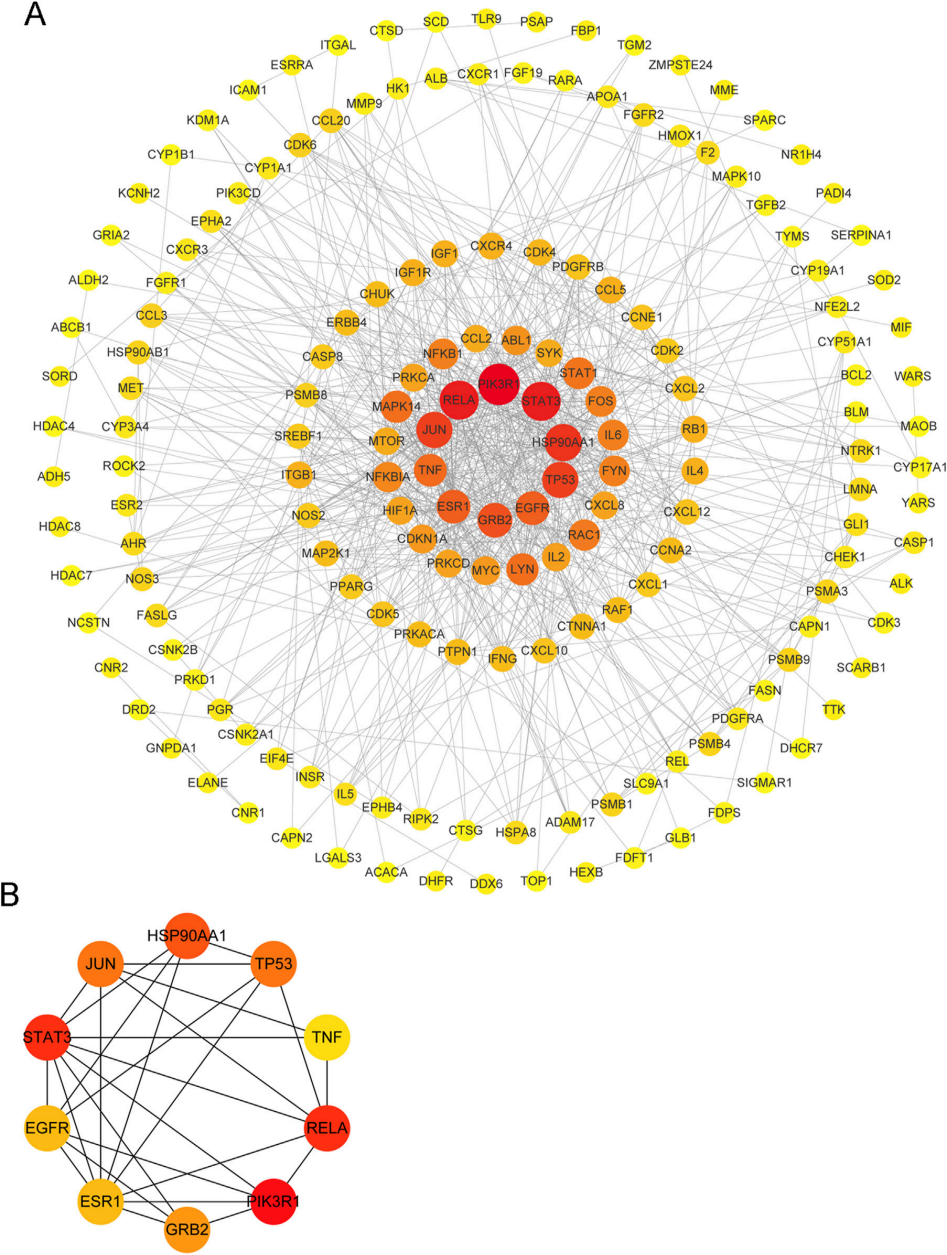
A protein-protein interaction (PPI) network of the target of *Candida albicans* infection treated with itraconazole and ritonavir via the autophagy pathway. **(A)** A PPI network of top 10° targets of autophagy therapy for *C. albicans* infection with itraconazole and ritonavir; **(B)** The interaction network of top 10 targets.

A PPI network containing 194 targets (proteins) was built, and the top 10-degree targets were selected as the core targets of autophagy therapy for *C. albicans* infection with itraconazole and ritonavir (Figure 2A). These targets were PIK3R1 (phosphoinositide-3-kinase regulatory subunit 1), TP53 (tumor protein P53), RELA (RELA proto-oncogene, NF-κB subunit), STAT3 (signal transducer and activator of transcription 3), HSP90AA1 (heat shock protein 90 alpha family class a member 1), GRB2 (growth factor receptor bound protein 2), EGFR (epidermal growth factor receptor), ESR1 (estrogen receptor 1), JUN (Jun Proto-Oncogene, AP-1 transcription factor subunit), and TNF (tumor necrosis factor) (Figure 2B).

### GO terms and pathways were enriched by 194 targets

**FIGURE 3:**
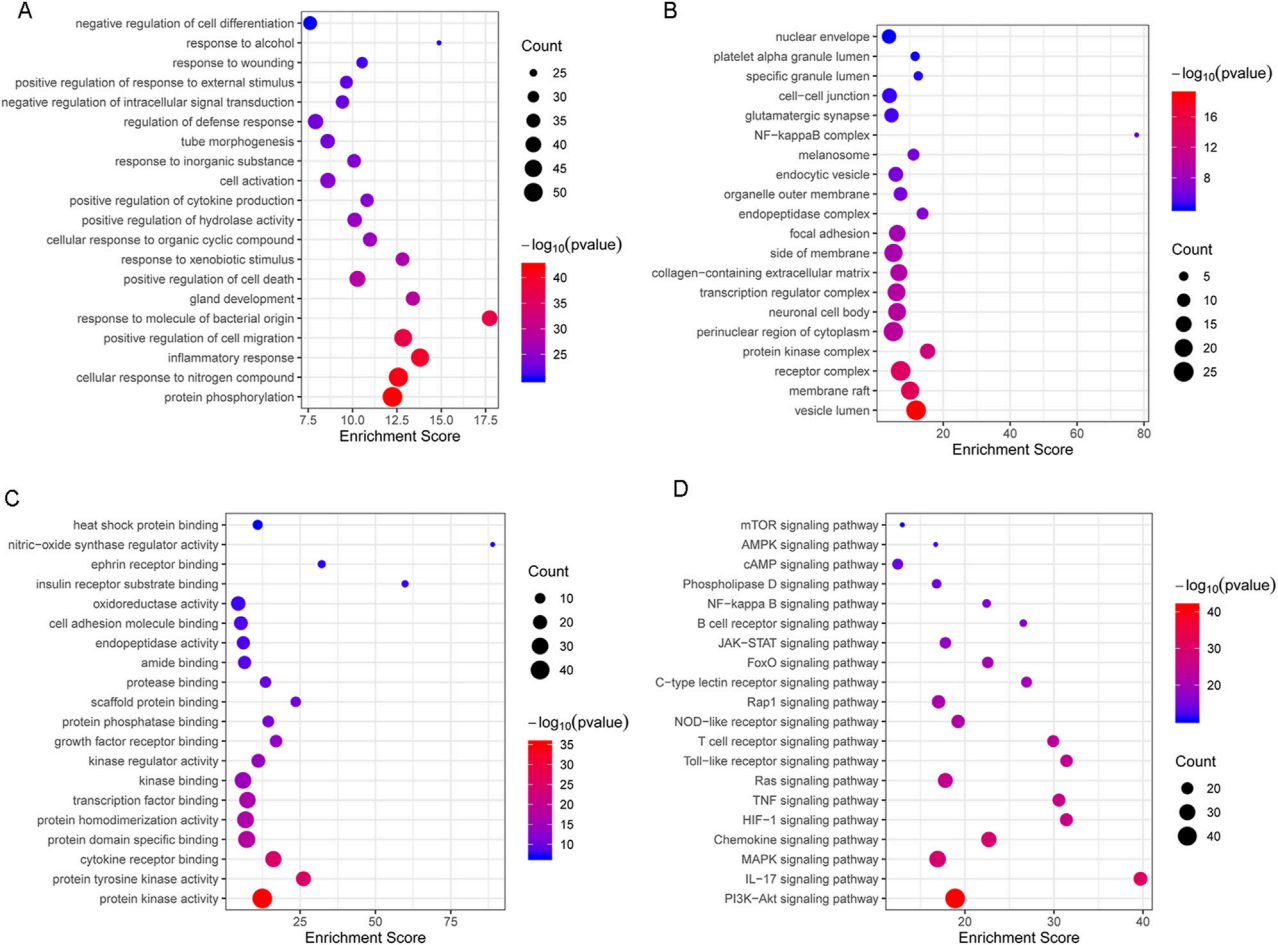
The top 20 GO (Gene Ontology)-biological process (BP), CC (cellular component), and MF (molecular function) terms were **(A–C)** and the KEGG enrichment analysis of targets involving in the treatment of *Candida albicans* infection with itraconazole and ritonavir by autophagy **(D)**.

The top 20 terms in three categories of BP, cellular component (CC), and molecular function (MF) were identified (Figures 3A–C). GO-(BP) terms were related to protein phosphorylation, cell response to nitrogen compounds, inflammatory response, positive regulation of cell migration, positive regulation of cell death, cell activation, and negative regulation of intracellular signal transduction (Figure 3A). KEGG enrichment analysis showed that the treatment of *C. albicans* infection with itraconazole and ritonavir via autophagy may be related to the pathways of PI3K-AKT, Il-17, MAPK, HIF-1, TNF, Ras, Toll-like receptor, JAK-STAT and NF-κB (Figure 3D).

### The drug-target-pathway-disease network was constructed

**FIGURE 4:**
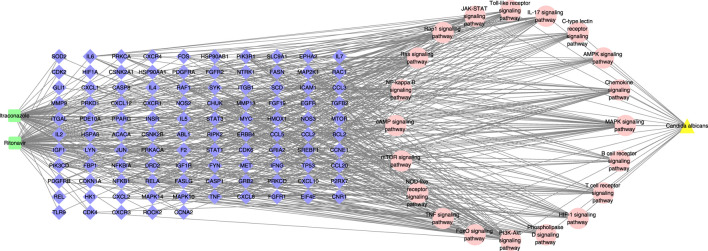
The drug-target-pathway-disease network. The green squares represent drugs, the blue diamonds represent targets, the pink circles represent pathways, and the yellow triangles represent diseases.

The drug-target-pathway-disease network in Figure 4 indicated the mapping relationship between the top 20 signal pathways related to *C. albicans* infection and their corresponding targets in KEGG enrichment analysis. According to the network analysis, most of the target genes were involved in two or more pathways, and the therapeutic effect of itraconazole and ritonavir in *C. albicans* infection has multicomponent and multitarget characteristics.

### Targets were well docked with itraconazole and ritonavir

**FIGURE 5:**
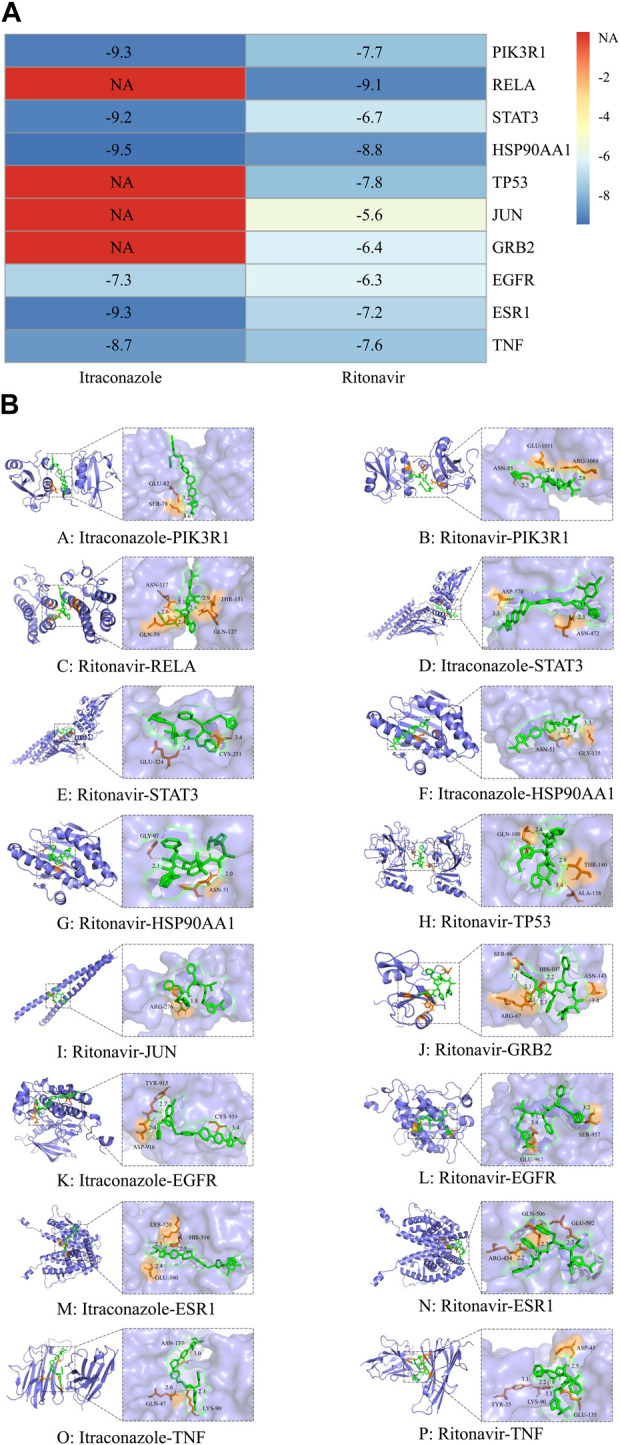
Heat map of molecular docking results of itraconazole and ritonavir corresponding target protein molecules **(A)**. The horizontal axis is the name of the drug and the vertical axis is the name of the target protein. The number in the grid represents the binding free energy (kcal/mol), the closer the color is to blue, the greater the absolute value of the binding energy, and the red part indicates that there is no correspondence between the drug and the target. Binding patterns of itraconazole and ritonavir and corresponding target proteins **(B)**. Blue is the protein structure, green is the drug structure, orange is the binding site, yellow is the hydrogen bond, and the text is the predicted drug protein binding site and hydrogen bond length.

The outcomes of molecular docking displayed that the binding energies of the top 10 core genes to the corresponding drugs were less than −5 kcal mol, and some of them were less than −7.0 kcal/mol. Itraconazole docked to PIK3R1 through SER-78 and GLU-82 with −9.3 kcal/mol of intermolecular binding energy, docked to STAT3 through asparagine (ASN)-472 and aspartic acid (ASP)-570 with −9.2 kcal/mol, docked to HSP90AA1 through ASN-51 and glycine (GLY)-135 with −9.5 kcal/mol, docked to EGFR through tyrosinase (TYR)-915, ASP-916 and cysteine (CYS)-939 with −7.3 kcal/mol, docked to ESR1 through glutamic acid (GLU)-380, histidine (HIS)-516 and lysine (LYS)-520 with −9.3 kcal/mol, and docked to TNF through glutamine (GLN)-47, LYS-90 and ASN-137 with −8.7 kcal/mol. Ritonavir docked to PIK3R1 through ASN-85, GLU-1011 and arginine (ARG)-1088 with −7.7 kcal/mol, docked to RELA through GLN-59, ASN-117, GLN-127 and threonine (THR)-131 with −9.1 kcal/mol, docked to HSP90AA1 through ASN-51 and GLY-97 with −8.8 kcal/mol, docked to TP53 through GLN-100, THR-140, alanine (ALA)-138 with −7.8 kcal/mol, docked to ESR1 through ARG-434, GLU-502 GLN-506 with −7.2 kcal/mol, and docked to TNF through TYR-35, ASP-45, LYS-90 and GLU-135 with −7.6 kcal/mol. This indicated that this compound has the potential to form a stable binding conformation to the target protein (Figure 5A). The docking mode was shown in Figure 5B. Molecular docking results suggested that these core targets may involve in the therapy of *C. albicans* infection with itraconazole and ritonavir.

### Molecular dynamics simulation of itraconazole and ritonavir with representative target proteins

**FIGURE 6:**
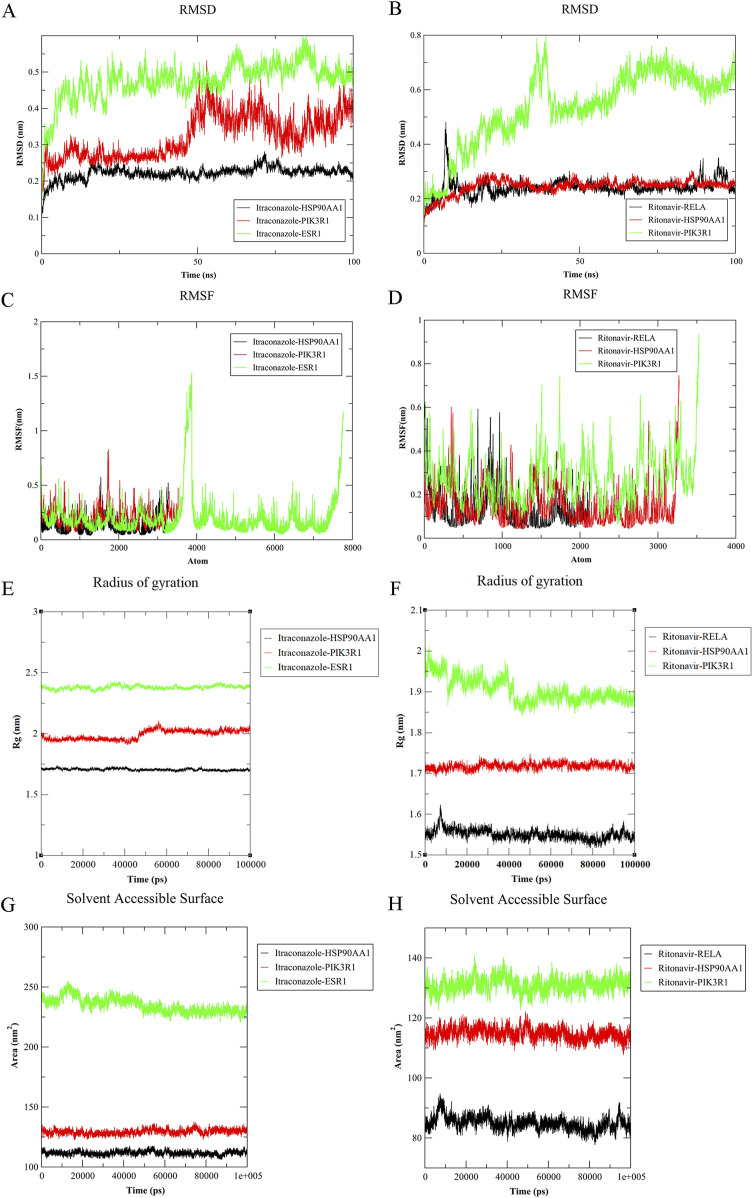
Molecular dynamics simulations of itraconazole and ritonavir with core target proteins. **(A)** RMSD analysis of 100 - ns simulation of itraconazole with HSP90AA1, PIK3R1, and ESR1; **(B)** RMSD analysis of 100 - ns simulation of ritonavir with RELA, HSP90AA1, and PIK3R1; **(C)** RMSF analysis of itraconazole with HSP90AA1, PIK3R1, and ESR1; **(D)** RMSF analysis of ritonavir with RELA, HSP90AA1, and PIK3R1; **(E)** Radius of gyration analysis of itraconazole with HSP90AA1, PIK3R1, and ESR1; **(F)** Radius of gyration analysis of ritonavir with RELA, HSP90AA1, and PIK3R1; **(G)** Solvent - accessible surface area analysis of itraconazole with HSP90AA1, PIK3R1, and ESR1; **(H)** Solvent - accessible surface area analysis of ritonavir with RELA, HSP90AA1, and PIK3R1.

The smaller the RMSD value and the milder the fluctuation, the more stable the binding of the complex. As shown in Figures 6A,B, the RMSD fluctuations of the complexes formed by itraconazole and HSP90AA1, PIK3R1, ESR1, and those formed by ritonavir and RELA, HSP90AA1, PIK3R1 were relatively small (<0.8 nm). Specifically, the RMSD of the complexes of itraconazole and HSP90AA1, ritonavir and RELA, HSP90AA1 fluctuates around 0.2, showing good convergence and stable binding.

RMSF reflects the flexibility of the system at the residue level. When ritonavir binds to HSP90AA1 and PIK3R1, the RMSF amplitudes were similar, and the positions of the RMSF peaks and valleys were basically the same. It is speculated that the binding sites of the two ligands to the receptor were similar, suggesting that the two monomers may interact to some extent to exert their activities (Figures 6C,D).

The results of RoG calculation were shown in Figures 6E,F. The complexes formed by itraconazole and its target proteins, and those formed by ritonavir and RELA or HSP90AA1 showed little fluctuation within 100 ns. Although the complex of ritonavir and PIK3R1 fluctuated in the initial stage, it became stable after 40 ns. Besides, during the simulation process, the protein did not experience any unfolding events.

The SASA results reflect the molecular size and the interaction with the environment. The complexes formed by itraconazole and ritonavir showed relatively small fluctuations, indicating a relatively stable protein structure. At 15 ns, a flexible region of ESR1 interacting with itraconazole may be transiently exposed (Figures 6G,H).

### The miRNAs of miR-486-5p, miR-411-5p. 1 and miR-296-5p were identified

**FIGURE 7:**
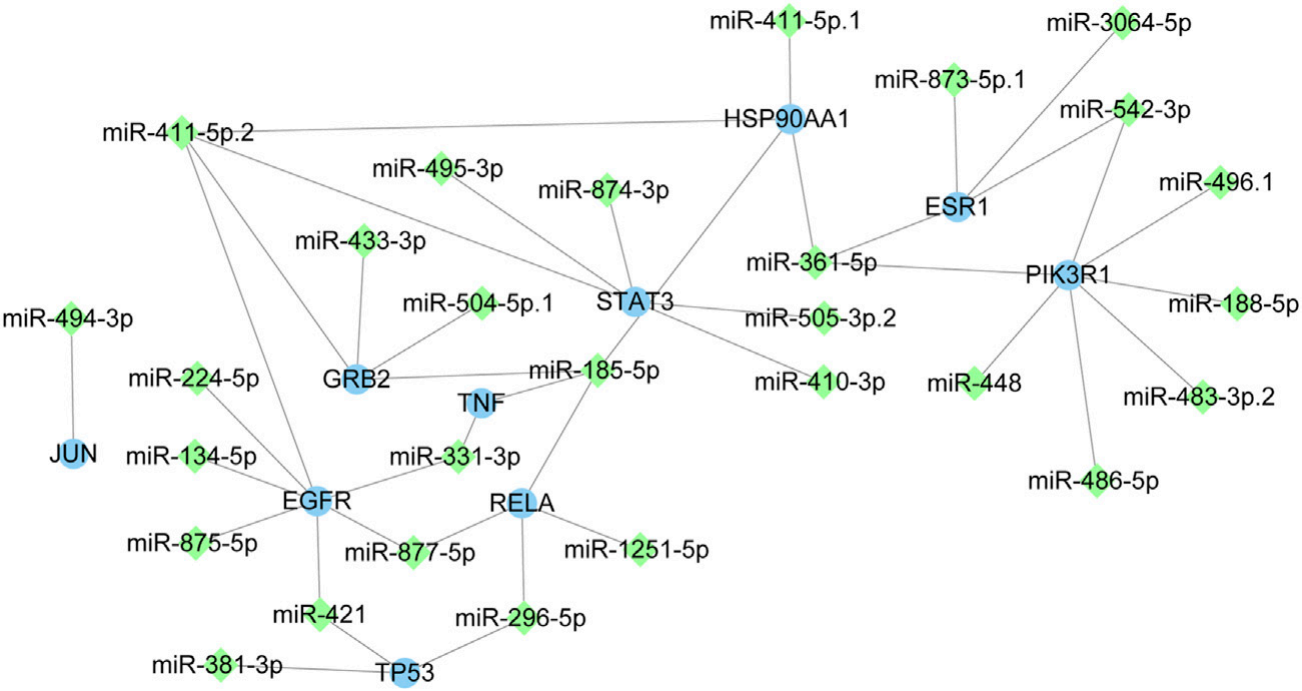
The interaction network of core targets and miRNAs. The blue circle represents targets and the green diamond represents miRNAs.

The core target-miRNA interaction network was constructed (Figure 7). Twenty-eight miRNAs were predicted for the 10 core targets. Among these miRNAs, miR-486-5p, miR-411-5p. 1 and miR-296-5p were the key miRNAs with highest context score percentile (score = 98, Table 2). MiR-486-5p could regulate PIK3R1, miR-411-5p.1 could regulate HSP90AA1 and miR-296-5p could regulate TP53 and RELA in response to *C. albicans* infection with itraconazole and ritonavir.

**TABLE 2:** The miRNAs that regulates core target proteins predicted by TargetScan.

Gene name	miRNA	Position in the UTR	Seed match	Context score percentile
PIK3R1	miR-486-5p	956–963	8mer	98
PIK3R1	miR-361-5p	2995–3002	8mer	87
PIK3R1	miR-188-5p	1564–1571	8mer	85
PIK3R1	miR-542-3p	1579–1586	8mer	82
PIK3R1	miR-496.1	1611–1618	8mer	80
PIK3R1	miR-483-3p.2	1877–1884	8mer	56
PIK3R1	miR-448	1907–1914	8mer	49
RELA	miR-185-5p	324–331	8mer	97
RELA	miR-1251-5p	1986–1993	8mer	95
RELA	miR-296-5p	1069–1076	8mer	93
RELA	miR-877-5p	341–348	8mer	92
STAT3	miR-874-3p	1609–1616	8mer	92
STAT3	miR-410-3p	2499–2506	8mer	91
STAT3	miR-495-3p	1892–1899	8mer	86
STAT3	miR-505-3p.2	1117–1124	8mer	80
STAT3	miR-411-5p.2	792–799	8mer	32
HSP90AA1	miR-411-5p.1	144–151	8mer	98
HSP90AA1	miR-361-5p	46–53	8mer	97
HSP90AA1	miR-185-5p	486–493	8mer	83
HSP90AA1	miR-411-5p.2	145–152	8mer	79
TP53	miR-296-5p	602–609	8mer	98
TP53	miR-421	643–650	8mer	95
TP53	miR-381-3p	981–988	8mer	94
JUN	miR-494-3p	988–995	8mer	64
GRB2	miR-433-3p	2137–2144	8mer	95
GRB2	miR-504-5p.1	1150–1157	8mer	95
GRB2	miR-185-5p	1787–1794	8mer	95
GRB2	miR-411-5p.2	741–748	8mer	80
EGFR	miR-134-5p	1580–1587	8mer	92
EGFR	miR-875-5p	270–277	8mer	91
EGFR	miR-224-5p	3122–3129	8mer	69
EGFR	miR-331-3p	2116–2123	8mer	54
EGFR	miR-421	3909–3916	8mer	49
EGFR	miR-877-5p	2949–2956	8mer	37
EGFR	miR-411-5p.2	4206–4213	8mer	32
ESR1	miR-873-5p.1	787–794	8mer	91
ESR1	miR-3064-5p	1102–1109	8mer	49
ESR1	miR-542-3p	1256–1263	8mer	44
ESR1	miR-361-5p	372–379	8mer	32
TNF	miR-331-3p	731–738	8mer	97
TNF	miR-185-5p	374–381	8mer	97

UTR, untranslated region.

## Discussion

*C. albicans* as a common opportunistic pathogen of human is life-threatening in severe cases (Sharma and Chakrabarti, 2023). Autophagy is involved in maintaining body homeostasis to prevent and respond to infectious diseases (Levine et al., 2011). Infection with *C. albicans* has been reported to induce increased expression of autophagy markers LC3, ATG5, and LAMP1, and study also showed that apoptosis and necrosis was reduced in human vaginal epithelial cells overexpressed with wild-type ATG5 (Shroff and Reddy, 2018; Shroff et al., 2018). Autophagy-related ATG5 and ATG16L1 have been shown to contribute to lysosomal exocytosis-mediated plasma membrane repair (Lapaquette et al., 2022). In addition, autophagy was involved in *C. albicans*-induced release of neutrophil traps (Kenno et al., 2016). Itraconazole and ritonavir has anti-*C. albicans* effects and can also associated with autophagy (Gibellini et al., 2012; Feng et al., 2021; Shen et al., 2021; Ming et al., 2022; Elkhoely et al., 2023a). This study exploring the potential mechanism of itraconazole combined with ritonavir in the treatment of *C. albicans* infection through autophagy pathway. The results may provide a new idea for clinical medication of *C. albicans* infection.

The core target screening and molecular docking analysis showed that itraconazole combined with ritonavir could treat *C. albicans* infection through multi-target and multi-pathway regulation of autophagy, several key miRNAs may be involved in it. A total of 194 potential autophagy-related targets were identified, and 10 core targets, such as PIK3R1, RELA, STAT3, HSP90AA1, TP53, might play roles in the treatment of *C. albicans* infection. PIK3R1, HSAP90AA1 and TP53 have been found to be novel autophagy-related proteins involved in the pathogenesis of diabetic retinopathy disease (Sarmah et al., 2023). RELA encodes the P65 subunit of NF-κB, and blocking NF-κB activity increases reactive oxygen species levels and induces autophagy in cell death (Laribee et al., 2023). STAT3 is involved in the expression of the autophagy-related gene ATG5 in Glioblastoma multiforme stem cells (Laribee et al., 2023). Whether itraconazole and ritonavir can modulate autophagy by regulating these predicted core targets in the treatment of *C. albicans* infection has not been clearly demonstrated. With the aim of further verifying the effect of core genes, we simulated the docking mode of drug and core targets. The results exhibited that the binding energy between the core target and the corresponding drug was less than −5 kcal/mol, this showed that the target protein and the corresponding compounds had good binding activity. Moreover, PIK3R1, HSP90AA1, ESR1, TNF with itraconazole and ritonavir, STAT3, EGFR with itraconazole, TP53, RELA with ritonavir have strong binding activity. It suggested that itraconazole and ritonavir may be effective in the treatment of *C. albicans* infection by interacting with core targets.

To further analyze the potential mechanisms of itraconazole and ritonavir for autophagy-related regulation in the treatment of *C. albicans* infection, we performed GO and KEGG enrichment analyses. GO-BP analysis showed that 194 potential targets were involved in protein phosphorylation, cell response to nitrogen compounds, inflammatory response, positive regulation of cell migration, and positive regulation of cell death. KEGG pathway analysis showed that itraconazole and ritonavir may participate in the treatment of *C. albicans* infection by regulating autophagy through PI3K-AKT, IL-17, MAPK, HIF-1, JAK-STAT and NF-κB signaling pathways. Notably, the PI3K-AKT signaling pathway with the highest count was enriched by 43 targets, and PIK3R1, RELA, HSP90AA1, TP53, GRB2, and EGFR were all enriched in this pathway. Studies have shown that the PI3K-AKT signaling pathway was activated after *C. albicans* infection, which may be related to the pathmechanism of *C. albicans* infection (Moyes et al., 2014). Inhibition of the PI3K-AKT signaling pathway has been shown to attenuate multiple types of inflammatory damage, such as diabetes-induced kidney damage, as well as alcohol-related pancreatitis (Gao et al., 2022). Furthermore, interference of the PI3K-AKT-mTOR signaling pathway facilitates autophagy in articular chondrocytes and depresses the inflammatory response in osteoarthritis rats, and activation of the PI3K-AKT pathway has been shown to inhibit autophagy and promote apoptosis of glioma cells (Xue et al., 2017; Chen H. et al., 2023). Itraconazole exerts therapeutic effects by inhibiting the PI3K-AKT pathway in a variety of diseases, and ritonavir has also been shown to block Akt signaling (Chen et al., 2019; Wang et al., 2020). Therefore, we speculate that itraconazole combined with ritonavir may involve in the treatment of *C. albicans* infection by promoting autophagy via PI3K-AKT pathway. Furthermore, mulberroside A can inhibit the MAPK, NF-κB, and PI3K-AKT-mTOR signaling pathways, and promote autophagic processes to exert cartilage protection (Lu et al., 2023). Inhibition of the NF-κB signaling attenuates the effect of PDCD4 knockdown on autophagy (Chen M. L. et al., 2023). Inhibition of IL-17 can increase the expression of STAT3 and HIF-1 α in scars and lead to autophagy deficiency, which can be reversed by HIF-1 α inhibitor (Lee et al., 2022). These results suggested that the itraconazole pathway is devoted to the regulation of autophagy, and the PI3K-AKT pathway may be instrumental in the anti-*C. albicans* response.

Although direct experimental validation has not been performed, our molecular docking and molecular dynamics simulations provide robust computational support for specific drug-target interactions. At present, computational biology has become an important tool for target prediction. For example, integrative approaches combining AlphaFold2 and molecular docking have become pivotal for guiding ligand discovery against challenging targets (Lyu et al., 2024). Molecular docking analysis indicated that itraconazole and ritonavir possess strong binding affinities with the cote target proteins, with binding free energies ranging from −5.6 to −9.5 kcal/mol. In the molecular dynamics simulation, flexible docking was carried out for itraconazole with HSP90AA1, PIK3R1 and ESR1, as well as ritonavir with RELA, HSP90AA1 and PIK3R1. This process lasted for 100 ns, and overall, the structures of the formed complexes remained stable throughout. This further corroborates the results of molecular docking, indicating their inherent high biological activity.

To further investigate the role of miRNA-mediated regulation of gene expression in autophagy-based treatment of *C. albicans* infection with itraconazole and ritonavir, a core target-miRNA interaction network was constructed. We found that miR-486-5p, miR-411-5p.1, and miR-296-5p were the most functional and key miRNAs that might be regulated during autophagy-based treatment of *C. albicans* infection with itraconazole combined with ritonavir. The predicted miRNAs may serve as upstream regulators of autophagy-related targets. Their potential role in fine-tuning drug efficacy or resistance mechanisms warrants further experimental validation, as dysregulated miRNA expression has been linked to fungal pathogenesis and host immune responses (Kimura et al., 2023). The identification of key miRNAs underscores the potential role of post-transcriptional regulation in the autophagic response to itraconazole and ritonavir. As shown in the network, miR-486-5p could regulate PIK3R1. As a key subunit of the PI3K-AKT pathway, PIK3R1 activates Akt/mTOR signaling to inhibit autophagy. The strong binding free energies between PIK3R1 and itraconazole (−9.3 kcal/mol) or ritonavir (−7.7 kcal/mol) suggests direct inhibition of PIK3R1 by these drugs, while miR-486-5p enhances this effect by post-transcriptionally silencing PIK3R1 through 3′UTR binding. This aligns with our KEGG analysis showing PI3K-AKT pathway enrichment and previous reports of miR-486-5p/PIK3R1 axis in non-small cell lung cancer, pancreatic cancer and diabetic nephropathy (Tian et al., 2019; Kong et al., 2020; Su et al., 2024). It has been shown that miR-486-5p inhibitor induces autophagy and enhances S-adenosyl-l-methionine-induced autophagic process by increasing PTEN gene expression and inhibiting Akt signaling (Ilisso et al., 2018). In hepatoma cells, inhibition of miR-411-5p reversed MIAT knockdown-triggered suppression of STAT3 and PD-L1 expression, and regulation of the autophagy-related gene STAT3 by miR-411-5p may also be involved in the therapy of *C. albicans* infection with itraconazole and ritonavir (Zhang et al., 2022). HSP90AA1 mediating autophagy contributes to chemotherapy resistance in osteosarcoma (Xiao et al., 2018). It is reported that a direct connection between HSP90AA1 and the AKT-mTOR pathway triggers autophagy, which is a critical step for controlling infection (Hu et al., 2015). miR-411-5p-mediated HSP90AA1 regulation may destabilize these proteins, leading to mTOR inactivation and autophagy induction. This is supported by study showing HSP90 inhibitors enhance autophagy in cancer cells (Lan et al., 2024). TP53 and RELA were identified to be regulated by miR-296-5p in the network. The tumor suppressor gene TP53 plays a key role in autophagy induction or inhibition by targeting DRAM or TIGAR, respectively (Hu et al., 2019). In high-concentration sodium fluoride intervention experiments, miR-296-5p may promote autophagy through the AMPK-ULK1 pathway (Luo et al., 2022). However, the regulation of HSP90AA1 by miR - 411 - 5p, as well as the regulation of TP53 and RELA by miR - 296 - 5p, has not been previously reported. These results suggest that miR-486-5p, miR-411-5p. 1 and miR-296-5p may participate in the mechanism of treatment of *C. albicans* with itraconazole and ritonavir through regulation of autophagy.

The primary targets of azole drugs (such as itraconazole) are sterols in the fungal cell membrane, especially lanosterol 14α - demethylase (Erg11), which disrupts membrane integrity by inhibiting ergosterol synthesis (Song et al., 2025). In recent years, studies have suggested that azole drugs may affect host proteins through multiple pathways. For example, changes in the cell membrane may trigger indirect effects, such as cellular stress, which in turn may affect the autophagy process. The HMGB1/TLR4 axis, the NF - κB signaling pathway (Elkhoely et al., 2023b), and lysosomal homeostasis (Marastoni et al., 2022) may also be involved in this process. Ritonavir, as an HIV protease inhibitor, has also been reported to indirectly regulate autophagy through the MAPK pathway (Chen et al., 2022). Evidently, azole drugs may interact with multiple targets in host cells through “off - target effects”, and the triazole ring and hydrophobic side chain in their structure may endow them with the potential to bind to non - sterol proteins. The indirect mechanism of “cell membrane perturbation → stress → autophagy activation” is an important supplementary perspective. Some drug interventions can induce endoplasmic reticulum stress to activate protective autophagy (Lebeau et al., 2017).

There are some limitations in our study. First, this study is the absence of direct experimental validation to confirm itraconazole/ritonavir binding to autophagy-related proteins in biological systems. While molecular docking and molecular dynamics simulations provide strong computational support, clinical translation requires further validation in *in vitro* and *in vivo* models. Additionally, the specific contributions of individual miRNAs (e.g., miR-486-5p) and the precise role of autophagy in drug synergy (e.g., fungal vs host cell targets) remain to be experimentally characterized. While our findings are inherently predictive, they provide a mechanistic foundation for hypothesis-driven experimentation. In subsequent research, experimental verification of these computational conclusions will be prioritized.

## Conclusion

PIK3R1, RELA, STAT3, HSP90AA1, TP53, multiple pathways, especially the PI3K-AKT pathway, and miRNAs of miR-486-5p, miR-411-5p. 1 and miR-296-5p were potentially involved in the therapeutic mechanism of autophagy in *C. albicans* infection treated with itraconazole and ritonavir. These *in silico* predictions, while requiring experimental validation, offer a mechanistic framework for understanding potential drug-host interactions. In the future study, the combination of itraconazole and ritonavir on *C. albicans* infection *in vivo* and *in vitro* need further investigation, and the roles of predictive targets, signal pathways and miRNAs in the treatment of *C. albicans* infection needed to be further explored.

## Data Availability

The original contributions presented in the study are included in the article/supplementary material, further inquiries can be directed to the corresponding author.

## Abbreviations

(PPI), protein-protein interaction; (BP), biological processes; (MeSH), Medical Subject Headings; (HADb), human autophagy database; (MSigDB), molecular signatures database; (GO), Gene Ontology; (KEGG), Kyoto Encyclopedia of Genes and Genomes; (PDB), Protein Data Bank; (CC), cellular component; (MF), molecular function; (ASN), asparagine; (ASP), aspartic acid; (GLY), glycine; (TYR), tyrosinase; (CYS), cysteine; (GLU), glutamic acid; histidine (HIS); (LYS), lysine; (GLN), glutamine; (ARG), arginine; (THR), threonine; (ALA), alanine.
